# Perforated Meckel's Diverticulum causing Intussusception in a Neonate

**DOI:** 10.21699/jns.v6i3.568

**Published:** 2017-08-10

**Authors:** Hamdi Louati, Mohamed Zouari, Mohamed Jallouli, Mahdi Ben Dhaou, Hayet Zitouni, Riadh Mhiri

**Affiliations:** Department of Pediatric Surgery, Hedi Chaker Hospital, Sfax, Tunisia

**DEAR SIR**

Intussusception is well-recognised in young children; however this condition is very rare in newborns [1]. Neonatal intussusception is usually caused by several conditions including Meckel’s diverticulum, intestinal polyps and intestinal duplication [1]. Meckel’s diverticulum can present at any age group; however rarely, a symptomatic Meckel’s diverticulum may present in neonates [2]. We report a rare case of neonatal bowel obstruction with an intussusception secondary to perforated Meckel’s diverticulum..

A male newborn at term, weighing 2350 grams, presented with bilious vomiting without abdominal distension. The plain abdominal radiograph showed a gastric distention without pneumatosis intestinalis or free air. Laboratory tests did not demonstrate any alteration. A midgut volvulus was suspected based on ultrasound data. Upon surgical exploration, we found an ileo-ileal intussusception which was manually reduced. After reduction, we founded an ileal perforated Meckel’s diverticulum (Fig.1). The affected bowel segment was resected and end-to-end ileo-ileal anastomosis was done. The postoperative course was uneventful. Histopathology report was consistent with perforated Meckel’s diverticulum. 

 Intussusception occurs very infrequently in the neonatal period, with a reported incidence ranging from 0.3% to 2.7% in the first month of life, and results in less than 3% of all neonatal bowel obstructions [3]. Neonatal intussusception does not have any classical radiological signs. The most common imaging findings in neonates with intussusception are signs of ileus such as dilation of bowel loops [4]. In our case radiological exploration was not helpful. Perforated Meckel’s diverticulum is rarely found in neonates [5]. To summarize, neonatal intussusception is an extremely rare clinical entity. This diagnosis is frequently missed and requires a high index of suspicion. 

**Figure:**
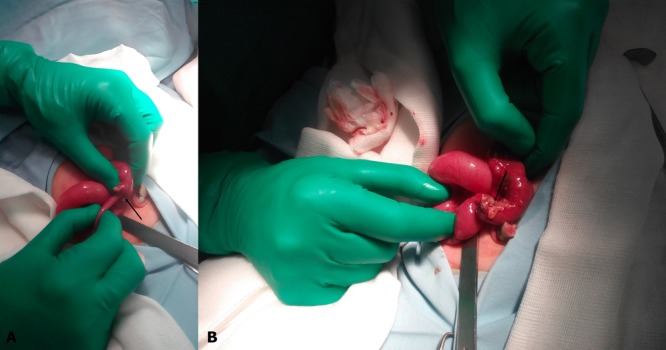
Figure 1: A: Intraoperative view showing the ileo-ileal intussusception (arrow). B: Intraoperative view, after reduction, showing the perforated Meckel’s diverticulum (arrow)

## Footnotes

**Source of Support:** None

**Conflict of Interest:** None

## References

[1] Avansino JR, Bjerke S, Hendrickson M. Clinical features and treatment outcome of intussusception in premature neonates. J Pediatr Surg. 2003;38:1818-21. 10.1016/j.jpedsurg.2003.08.048 14666476

[2] Sinha CK, Fishman J, Clarke SA. Neonatal Meckel's diverticulum: spectrum of presentation. Pediatr Emerg Care. 2009;25:348-9. 10.1097/PEC.0b013e3181a34936 19444035

[3] Margenthaler JA, Vogler C, Guerra OM. Pediatric surgical images: small bowel intussusception in a preterm infant. J Pediatr Surg. 2002;37:1515-7. . 10.1053/jpsu.2002.35437 12378473

[4] Loukas I, Baltogiannis N, Plataras C. Intussusception in a premature neonate: A rare often misdiagnosed cause of intestinal obstruction. Case Rep Med. 2009;2009:607989. 10.1155/2009/607989 PMC2797753 20049335

[5] Oyachi N, Takano K, Hasuda N, et al. Perforation of Meckel's diverticulum manifesting as aseptic peritonitis in a neonate: report of a case. Surg Today. 2007;37:881. 10.1007/s00595-007-3519-3 17879039

